# Ramipril and ketogenic diet response in cognitive dysfunction of insulin-resistant rats

**DOI:** 10.3389/fphar.2025.1620139

**Published:** 2025-07-11

**Authors:** Nancy M. Abdel-Kareem, Shimaa M. Elshazly, May A. Abd El Fattah, Sawsan A. Zaitone, Thanaa A. Elmasry, Asmaa Saleh, Enas A. Abd El-Haleim

**Affiliations:** ^1^ Department of Pharmacology and Toxicology, Faculty of Pharmacy, Sinai University—Arish Branch, Arish, Egypt; ^2^ Department of Pharmacology and Toxicology, Faculty of Pharmacy, Zagazig University, Zagazig, Egypt; ^3^ Department of Pharmacology and Toxicology, Faculty of Pharmacy, Cairo University, Cairo, Egypt; ^4^ Department of Pharmacology and Toxicology, Faculty of Pharmacy, University of Tabuk, Tabuk, Saudi Arabia; ^5^ Department of Pharmacology and Toxicology, Faculty of Pharmacy, Suez Canal University, Ismailia, Egypt; ^6^ Department of Pharmacology and Toxicology, Faculty of Pharmacy, Tanta University, Tanta, Egypt; ^7^ Department of Pharmaceutical Sciences, College of Pharmacy, Princess Nourah bint Abdulrahman University, Riyadh, Saudi Arabia

**Keywords:** insulin resistance, ramipril, cognitive dysfuntion, ketogenic diet, normal diet

## Abstract

**Introduction:**

High fructose consumption induces insulin resistance (IR), which impairs cognitive functions. Recent studies have recommended the use of ramipril for the treatment of neurological disorders. In the current study, the effects of ramipril on cognitive dysfunction were compared in IR rats fed either a ketogenic diet (KD) or normal diet (ND).

**Methods:**

Fructose (10%) dissolved in drinking water was administered to the rats for 8 weeks to induce experimental IR. Ramipril (2 mg/kg daily; p.o.) was administered along with the ND or KD for an additional 5 weeks. Cognitive dysfunction was assessed at the end of the experiment using the Morris water maze (MWM) test. One-way and two-way analyses of variance (ANOVA) were used for comparisons.

**Results:**

The IR + ND group—as a diet control group—displayed a significant improvement in IR at the end of week 13 (1.63 ± 0.12 vs. 1.35 ± 0.06 in normal rats), as determined through the homeostasis model assessment of IR. Furthermore, brain-derived neurotrophic factors, lipid profile, insulin-degrading enzyme activities, and glycogen synthase kinase-3β activity were significantly ameliorated. The IR + KD and IR + ND + ramipril groups did not show significant improvements in most of the measured parameters compared to the normal and IR + ND groups. Notably, the IR + ND + ramipril group demonstrated significantly reduced tau protein and amyloid β (Aβ) levels. Differently, the IR + KD + ramipril group displayed ameliorated metabolic parameters (e.g., the IR index was 1.74 ± 0.13 vs. 3.34 ± 0.28 in the IR + ND + ramipril group and that of serum triglycerides (TGs) was 58.17 ± 1.85 vs. 97.5 ± 2.09 in the IR + ND + ramipril group), with no improvement in the cognitive function parameters.

**Discussion:**

Ramipril may be best indicated for the treatment of KD because of its preferable peripheral and central effects. However, KD may be administered for a while as it can treat accumulated Aβ and tau proteins, and patients must be aware of its adverse effects.

## 1 Introduction

In addition to the peripheral effects of insulin, including adjusting blood glucose levels, enhancing glycogenesis, decreasing lipid catabolism, and modulating inflammation, circulating insulin can cross the blood–brain barrier (Vargas et al., 2022). High insulin levels in the hypothalamus, pons, and medulla suggest that insulin is biosynthesized in brain cells (Blazquez et al., 2014). Insulin has a significant effect on the brain, not only in regulating glucose homeostasis but also in controlling food intake, centrally managing whole-body temperature, recognizing different objects, and processing sensory information (Cui et al., 2022). Insulin resistance (IR) was first described by Yalow and Berson in the 1960s. Several studies have focused on investigating the impact of IR on several diseases. As IR prevalence ranges from 15.5% to 46.5%, recent studies have investigated its negative effects on cognitive dysfunction (Shankpal and Surve, 2020). IR is identified as the insensitivity of insulin receptors to the circulating insulin and is characterized by certain metabolic syndromes presented as impaired glucose metabolism, hyperinsulinemia, hyperlipidemia, and obesity (Cui et al., 2022; Kim and Feldman, 2015). Genetic and environmental factors, such as stress and smoking, contribute to the incidence of IR (Cui et al., 2022). When IR persists for a long time, particularly centrally, it impairs neural and cognitive functions (Blazquez et al., 2014). The insulin-degrading enzyme (IDE) has a higher affinity for insulin than for amyloid-beta (Aβ). Hence, it reduces high insulin levels, leading to the accumulation of Aβ (Tian et al., 2023).

Although fructose, a natural sugar found in ingested honey and fruits (Gomez-Pinilla et al., 2021), is similar to glucose in its chemical structure, it does not increase blood glucose levels compared to other carbohydrates (Merino et al., 2019). However, evidence-based studies have demonstrated that poor eating habits correlated with the consumption of high fructose in the daily diet induce cognitive dysfunction, as the hippocampus, which plays a crucial role in learning and memorization, is highly sensitive to high fructose levels (Ross et al., 2009; Reichelt et al., 2018). Several studies suggested more than one mechanism explaining how fructose overconsumption induces cognitive dysfunction, including impairing oxidative metabolism and mitochondrial function, increasing oxidative stress and inflammatory mediator levels, and decreasing neurotrophic factor expression. These factors interrupt synaptic plasticity and cell metabolism, causing neural malfunction (Cui et al., 2022).

The ketogenic diet (KD) was first indicated for the treatment of refractory epilepsy, and its effects are promising in neural disorders. Recently, the KD has gained worldwide popularity, owing to its ability to reduce body weight in certain populations (Davis et al., 2021). Individuals who follow a KD utilize ketone bodies as the primary fuel source of energy instead of glucose, producing a few reactive oxygen species; therefore, the KD is considered an antioxidant diet. Notably, neurons favorably use ketone bodies as a source of energy because they do not alter their metabolism, unlike the glucose metabolism (Davis et al., 2021).

Antihypertensive drugs may ameliorate cognitive dysfunction in patients with hypertension. Observational studies have reported improvements in psychomotor function, speed, attention, and memory. Ramipril, an antihypertensive drug, is one of the angiotensin-converting enzyme inhibitor drugs (ACE-Is) (Wharton et al., 2012). Studies published by the Cardiovascular Health Study have suggested that centrally acting ACE-Is can recover cognitive function (Ouk et al., 2021).

Because the ability of ramipril to improve IR has been documented in diabetic patients and KD is currently recommended for treating IR, this experimental study was designed to compare the impact of ramipril on cognitive dysfunction in IR rats fed either a KD or a normal diet (ND). This experiment demonstrated how the KD may enhance the response to ramipril and reduce cognitive dysfunction in IR rats.

## 2 Methods

### 2.1 Animals and housing conditions

Adult male Wistar rats with body weights of 130–180 g were allowed to acclimatize to housing conditions for 2 weeks. Three rats were housed in each cage and kept at 28°C ± 2°C, under a standard day–night cycle without restricted access to food and water.

### 2.2 Drugs and chemicals

The Safety Company (Cairo, Egypt) provided fructose in the powdered form. Pharco Pharmaceuticals (Alexandria, Egypt) donated ramipril, which was mixed with polyethylene glycol-400 (PEG-400) (Al-Gomhoria Company, Cairo, Egypt).

We followed the guidelines of the American Institute of Nutrition (AIN-93M) to prepare KD. These include protein (casein: 142.09 and L-cysteine: 4.887), carbohydrates (dextrin only in 30), and fats (lard: 187.8, butter: 406, and soybean oil: 114.03) (Abdel-Kareem et al., 2024). The classic KD is a nutritional protocol based on the ingestion of a significant amount of fat (70%–80% of energy requirements from dietary fat), with a concomitant low supply of protein (approximately 15%–20% energy requirements from dietary protein) and a very low supply of carbohydrates (approximately 5%–10% energy requirements from dietary carbohydrates) (Zhu et al., 2022; Ashtary-Larky et al., 2022).

The other chemicals used during the experiment were of the highest offered pharmaceutical grade.

### 2.3 Induction of insulin resistance in rats

In brief, IR was induced by feeding rats with 10% fructose in drinking water (Gad et al., 2016); this continued for 8 weeks. An oral glucose tolerance test (OGTT) was performed on animals expected to suffer from IR, and only those animals were selected to complete the study.

At the end of the eighth week, the rats were fasted overnight. Fasting blood glucose (FBG) levels were measured. Subsequently, the rats were administered 2.5 mg per kg of glucose by oral gavage, and serum blood glucose levels were assayed pre- and post-administration of oral glucose at 30, 60, and 90 min (Ngakou Mukam et al., 2023). The blood glucose levels were detected using blood samples from the rats’ tails with a glucometer and Accu-Chek^®^ Performa test strips.

### 2.4 Experimental design

Thirty adult male Wistar rats were randomly distributed into five groups (six rats each) (Wei et al., 2024; Hattori et al., 2009), with a trial to reduce the total number of the used rats in the experiment, to complete the necessary statistical requirements. The rats were divided into the following groups:

Group A: normal control rats (non-diseased rats): rats received PEG 400 (ramipril vehicle) for 5 successive weeks by oral gavage (weeks 9–13).

Group B: (IR + ND): rats were fed 10% fructose for 8 weeks to induce IR (Gad et al., 2016); ND was continued for 5 more weeks (weeks 9–13).

Group C: (IR + KD): IR rats were fed the KD for 5 weeks (weeks 9–13) (Irfannuddin et al., 2021).

Group D: (IR + ND + ramipril): IR rats, fed with ND, received ramipril (2 mg/kg/day, orally) for 5 weeks (weeks 9–13) (Saager et al., 2020). The ramipril dose was non-hypotensive.

Group E: (IR + KD + ramipril): IR rats were fed the KD and received daily ramipril doses for 5 weeks (weeks 9–13).

A behavioral test was performed 1 day after the last dose of ramipril. Body mass was recorded weekly, and the net ratio change in the body weight throughout the experiment was calculated for each rat.

### 2.5 Behavioral test: Morris water maze (MWM) test

The Morris water maze (MWM) test is considered a practical test that is frequently used in research. The MWM test evaluated cognitive function in rats on the 86th day from the start of the experiment and was completed in 5 days. It was conducted in a circular white inner-walled pool with a depth of 50 cm and a diameter of 150 cm. The pool floor is divided into four quadrants: northeast, southeast, northwest, and southwest. A rounded platform, 15 cm in diameter and 30 cm in height, was positioned in each of the four quadrants throughout the experiment. The pool was filled with water at an appropriate temperature (22°C–25°C). The water was dyed with a powdered dye to mask the platform underneath the water surface by 1 cm. The pool walls had fixed objects during the test to provide spatial assistance to the animals. MWM was divided into acquisition and probe phases (Shang et al., 2022; Tahmasebi et al., 2020; Wang et al., 2021).

#### 2.5.1 Acquisition phase (short-term memory)

The first phase (acquisition phase) was performed in four trials over 4 successive days. Each rat was given 120 s per trial from each of the four quadrants to reach the hidden platform, and the time taken to reach the stage was recorded. The rats were left on the stage for 10 s. The rats were directed to the stage if they did not reach it within 120 s, and the timer was stopped for 10 s. The time was recorded as 120 s.

#### 2.5.2 Probe phase (long-term memory)

The stage was removed on the fifth day, and the rats swam for 60 s only. Hence, the interval spent during quadrant targeting was recorded for each rat.

The animals were euthanized on the 92nd day by cervical dislocation under anesthesia (30 mg/kg, i. p. of 2.5% thiopental sodium) (Vogler and Suckow, 2006). Each brain was excised and washed with saline, and the hippocampus was separated for estimating biochemical parameters. Histological and immunohistochemical investigations were performed using hippocampal sections. Dead animals’ bodies were stored in a −80°C deep freezer until incineration, following the recommended protocols of the Ethics Committee at the Faculty of Pharmacy, Cairo University (experimental code: PO341, date: 18 January 2021).

### 2.6 Biochemical assessment

Rats were fasted for 12 h after the probe phase of the behavioral test to collect blood samples from the retro-orbital plexuses using an anticoagulant (heparinized capillary tubes) in plain tubes, and samples were allowed to stand for up to 20 min before being centrifuged at 4,000 rpm for 15 min to separate the serum. The serum was separated using a micropipette in Eppendorf tubes and instantly preserved at −80°C until the bioassays were performed (Gad et al., 2016; Idowu et al., 2022; Mohammad et al., 2023).

#### 2.6.1 Homeostatic model assessment of insulin resistance (HOMA-IR) index

Fasting blood glucose levels were detected in the serum using a colorimetric assay kit for glucose (Sigma-Aldrich, St. Louis, Missouri, United States). Fasting insulin (FINS) was detected using a rat insulin Enzyme-Linked Immunosorbent Assay (ELISA) Kit from MyBioSource (MBS281388; San Deigo, Southern California, United States) by applying the following equation:

HOMA-IR index=(FBG(in mg/dL)×FINS(in μIU/mL)/405)

(Pitea et al., 2009), which has been used in several animal studies (Abdel-Mottaleb et al., 2022; Zaitone et al., 2011).

#### 2.6.2 Lipid profile assessment

The lipid profile in serum samples was assessed using a cholesterol kit (cat. no. Z5030055; BioChain, Hayward, CA, United States), high-density lipoprotein (HDL) and triglyceride (TG) kits (cat. no. 5603-01; XpressBio, Frederick, Maryland, United States), and a low-density lipoprotein (LDL) kit (cat. no. Z5030057; BioChain).

A glycogen synthase kinase-3 beta (GSK3β) kit (MBS909078), a brain-derived neurotrophic factor (BDNF) kit (MBS355345), and an IDE kit (MBS722683) were purchased from MyBioSource (San Diego). The activities of these enzymes were evaluated in hippocampal tissue homogenates by ELISA using an ELISA reader (TECAE, A 5082), following the kit’s instruction.

### 2.7 Histopathological examination

The histopathological data were analyzed by a histopathologist in a blinded manner to remove any bias. The cerebral cortex and hippocampal tissues were fixed for 2 days in neutral buffered formalin at 10%, inserted in paraffinized blocks, and dried for the histological and immunohistochemical studies, respectively. The sections were cut into 5 µm widths using a rotatory microtome and mounted on glass slides (Matsunami Glass Ind., Osaka, Japan). Hematoxylin and eosin (H&E) staining was used to stain the sections for histological studies (Gad et al., 2016; Idowu et al., 2022; Mokhtar et al., 2024; Ateyya et al., 2024).

### 2.8 Immunohistochemical analysis

The selected paraffinized blocks were cut into 4-µm-thick slices for immunohistochemistry. The slides were incubated with anti-β amyloid (catalog# A17911) and anti-tau (catalog# A1103) antibodies, which were purchased from ABclonal (Woburn, MA, United States). This was followed by incubation with suitable secondary antibodies (PI 0207, Rev. G DCN: 3129; Bio SB, Santa Barbara, California, United States). The slides were counterstained with hematoxylin for 30 s, followed by dehydration and mounting (Attia et al., 2023).

JPEG images were captured using an Olympus BX 40 light microscope equipped with an Olympus DP71 camera at a magnification of ×20 for the hippocampus and ×40 for the cerebral cortex. Positive neurons were counted, the net percentage of extracellular deposits in three high-power fields was determined using ImageJ software v1.54g (Schneider et al., 2012), and the mean ± standard error (S.E.) was calculated.

### 2.9 Statistical analysis

Records are reported as means ± S.E. Statistical investigation of all results is reported as the mean (*n* = 6) (Wei et al., 2024; Hattori et al., 2009). Statistical significance was tested using one-way and two-way analyses of variance (ANOVA), and *post hoc* comparisons were performed using the Tukey–Kramer test. All statistical tests were performed using GraphPad Prism (*P* < 0.05).

## 3 Results

### 3.1 Effect of a normal or ketogenic diet with or without ramipril treatment on body weight

**FIGURE 1:**
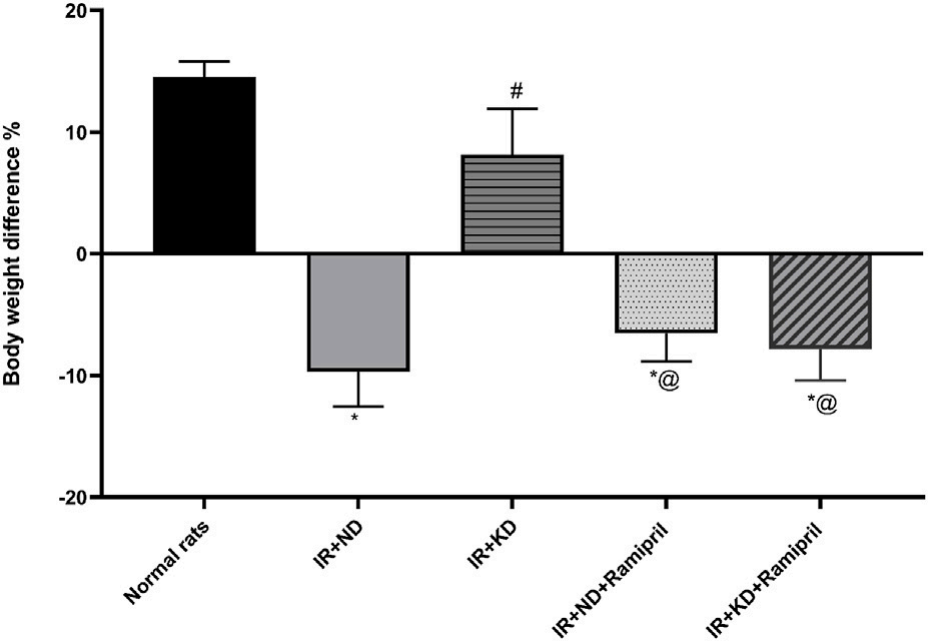
Normal diet (ND) and ketogenic diet (KD) with or without ramipril (2 mg/kg/day, orally) affect the body weight changes. Body weight difference (delta BWt) = the final weight of the insulin-resistant rats by the end of the experiment (end of week 13) − their weights at the end of week 8(end of the 10% fructose regimen). The IR + ND, IR + ND + ramipril, and IR + KD + ramipril groups showed significant decreases in body weight difference compared to the normal group; however, the IR + KD group showed no difference compared to the normal group. Data are analyzed using one-way ANOVA, followed by the Tukey–Kramer test, and represented as mean ± S.E. (*n* = 6 in each group), at *p < 0.05 vs. normal rats, ^#^p < 0.05 vs. IR + ND, and ^@^p < 0.05 vs. IR + KD.

As shown in Figure 1, unlike normal rats, a noticeable reduction was observed in the total body weight of rats in the IR + ND group. However, the IR + KD group showed a marked increase in body weight compared with the IR + ND group. In contrast, both ramipril IR groups fed either the ND or KD exhibited a noticeable decrease in body weight compared with both the normal and IR + KD groups, as shown in Figure 1 and Table 1.

**TABLE 1:** Results of the normal diet and ketogenic diet either with or without ramipril (2 mg/kg, orally) on the weight of inulin-resistant rats.

Investigational group	Delta body weight at the end of week 8	Percentage	Delta body weight at the end of week 13	Percentage
Normal rats	101	93	31	15
IR + ND	213	113	−45	−11
IR + KD	194	113	41	11
IR + ND + ramipril	247	151	−30.6	−8
IR + KD + ramipril	242	157	−31	−8

ND, normal diet; KD, ketogenic diet; IR, insulin resistance. Difference in body weight was assessed as follows: the mean body weight after 8 weeks of treatment with 10% fructose − the original body weight. Then, we calculated the difference in body weight and the percent change in final weight after ramipril and diet administration. Body weight was assessed as follows: body weight at the end of the study − body weight at the end of week 8. Afterward, the percentage change in weight was calculated for each rat.

### 3.2 Oral glucose tolerance test showed insulin resistance induced by receiving 10% fructose in drinking water

**FIGURE 2:**
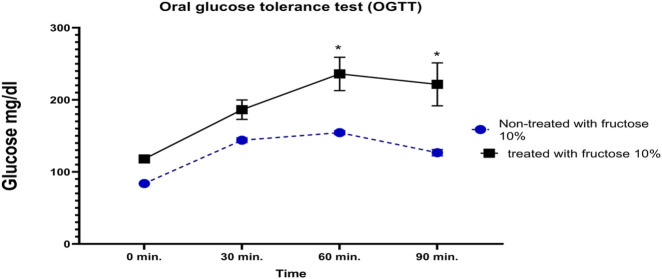
Impact of receiving 10% fructose for 8 weeks on the oral glucose tolerance test (OGTT). Blood glucose levels were evaluated pre- and post-receiving 2.5 mg/kg of glucose at different time points (0, 30, 60, and 90 min). The IR rats showed significant increases in blood glucose levels at 60 min and 90 min after glucose administration; however, the normal rats did not show similar elevations. Data are analyzed using two-way ANOVA, followed by the Bonferroni test for multiple comparisons, and represented as mean ± S.E (n = 6), at *p < 0.05 vs. normal rat control.

Blood glucose levels were measured before and after the administration of glucose (2.5 mg/kg) to confirm the induction of IR. A comparison of the experimental groups that received 10% fructose with the untreated group indicated a negligible increase in blood glucose levels after 0 or 30 min; however, a marked increase was detected in IR control rats after 60 and 90 min compared to the normal rats (Figure 2).

### 3.3 Ramipril normalized the increased fasting blood glucose levels, FINS levels, and HOMA-IR index induced by 10% fructose in drinking water followed by KD administration

The HOMA-IR index was estimated to assess the effect of ramipril and KD on IR. A significant increase in FBG, FINS, and the HOMA-IR index was detected in the IR + KD group compared with the normal group (Table 2). Although the FBG levels and HOMA-IR index of the IR + ND + ramipril group were significantly higher than those of the normal rats and the IR + ND and IR + KD groups, they were normalized. A similar effect was observed, together with the normalization of FINS levels, following ramipril administration with the KD.

**TABLE 2:** Effect of the normal diet and ketogenic diet with or without ramipril (2 mg per kilogram) on fasting blood glucose, fasting insulin, and the HOMA-IR index in insulin-resistant rats.

Experimental group	FBG	FINS	HOMA-IR index
Normal rats	70 ± 1.33	7.8 ± 0.16	1.35 ± 0.06
IR + ND	77 ± 2.77	8.55 ± 0.30	1.63 ± 0.12
IR + KD	85 * ± 2.72	10.58 *^a^ ± 0.66	2.22 * ± 0.23
IR + ND + ramipril	111 *^a^@ ± 3.39	12.17 *^a^ ± 0.73	3.34 *^a^@ ± 0.28
IR + KD + ramipril	78 + ± 2.23	9.03 + ± 0.34	1.74 + ± 0.13

ND, normal diet; KD, ketogenic diet; FBG, fasting blood glucose; FINS, fasting insulin; HOMA-IR index, homeostatic model assessment of insulin resistance index. Influence of ramipril (2 mg/kg) was tested either with or without a KD. Data were analyzed using one-way ANOVA, followed by the Tukey–Kramer test, and expressed as mean ± S.E (n = 6), at *p < 0.05 vs. normal rats.

^a^ p < 0.05 vs. IR + ND.

^@^ p < 0.05 vs. IR + ketogenic diet.

^+^ p < 0.05 vs. IR + ND + ramipril group.

### 3.4 Ketogenic diet affects cognitive function in the Morris water maze behavioral test

**FIGURE 3:**
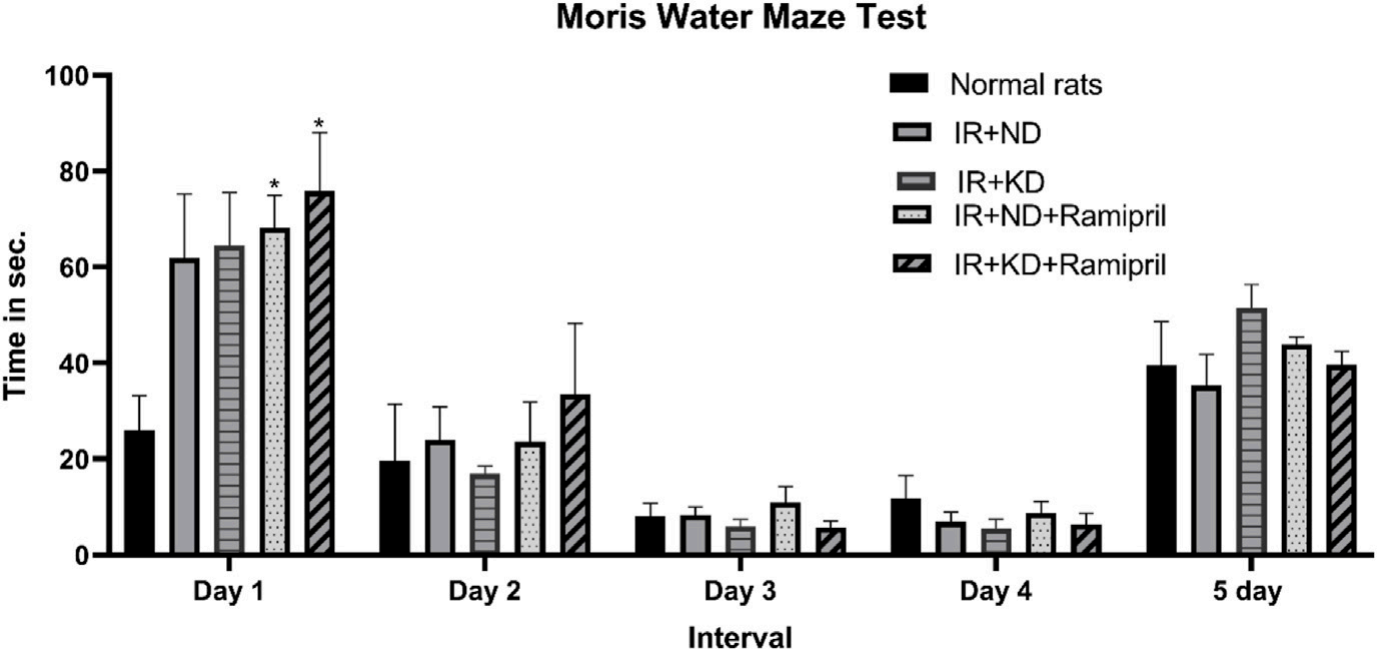
Effect of the ND and KD either with or without ramipril (2 mg/kg/day, orally) on the behavioral response in (IR) rats on the 86th day of the experiment. The Morris water maze test was used to test the time (in seconds) spent to arrive at the platform for 5 days. The results from the first day of experimentation indicated a prolonged time to reach the platform in IR rats that received ramipril. However, the other experimentation days (days 2–5) did not show similar differences. Records are analyzed using two-way ANOVA, followed by the Tukey–Kramer test, and represented as mean ± S.E (n = 6), at *p < 0.05 vs. normal rats.

Investigations on day 1 during the acquisition phase (short-term memory) indicated that both groups received ramipril and required a considerably longer period to reach the escape stage than normal rats. Furthermore, all groups demonstrated no significant differences on the subsequent days. No significant differences were observed between experimental groups during the probe phase (long-term memory) (Figure 3).

### 3.5 Blood lipid profile test

The levels of cholesterol, LDL, HDL, and triglycerides (TGs) were assayed in the serum to assess the effect of ramipril on IR-induced lipid dysregulation in ND and KD mice. ND administration after IR induction failed to restore normal HDL levels as a noteworthy decrease was noted in HDL levels compared to the normal group. However, the cholesterol, LDL, and TG levels were normalized after ND administration. These parameters were markedly worse in the IR + KD group than in the normal and IR + ND groups. The HDL levels were markedly lower in the IR + KD group than in the normal and IR + ND groups. In contrast, the administration of IR + ND + ramipril failed to improve the lipid profile, as the serum cholesterol, LDL, and TG levels were significantly high.

Simultaneously, HDL levels were markedly reduced in the IR + ND + ramipril group compared to those in the normal, IR + ND, IR + KD, and IR + KD + ramipril groups. Remarkable results were observed in the IR + KD + ramipril group; we noticed a major change in the levels of each lipid profile item. A marked reduction was observed in cholesterol levels in the IR + ND group compared to those in the IR + KD and IR + ND + ramipril groups.

**FIGURE 4:**
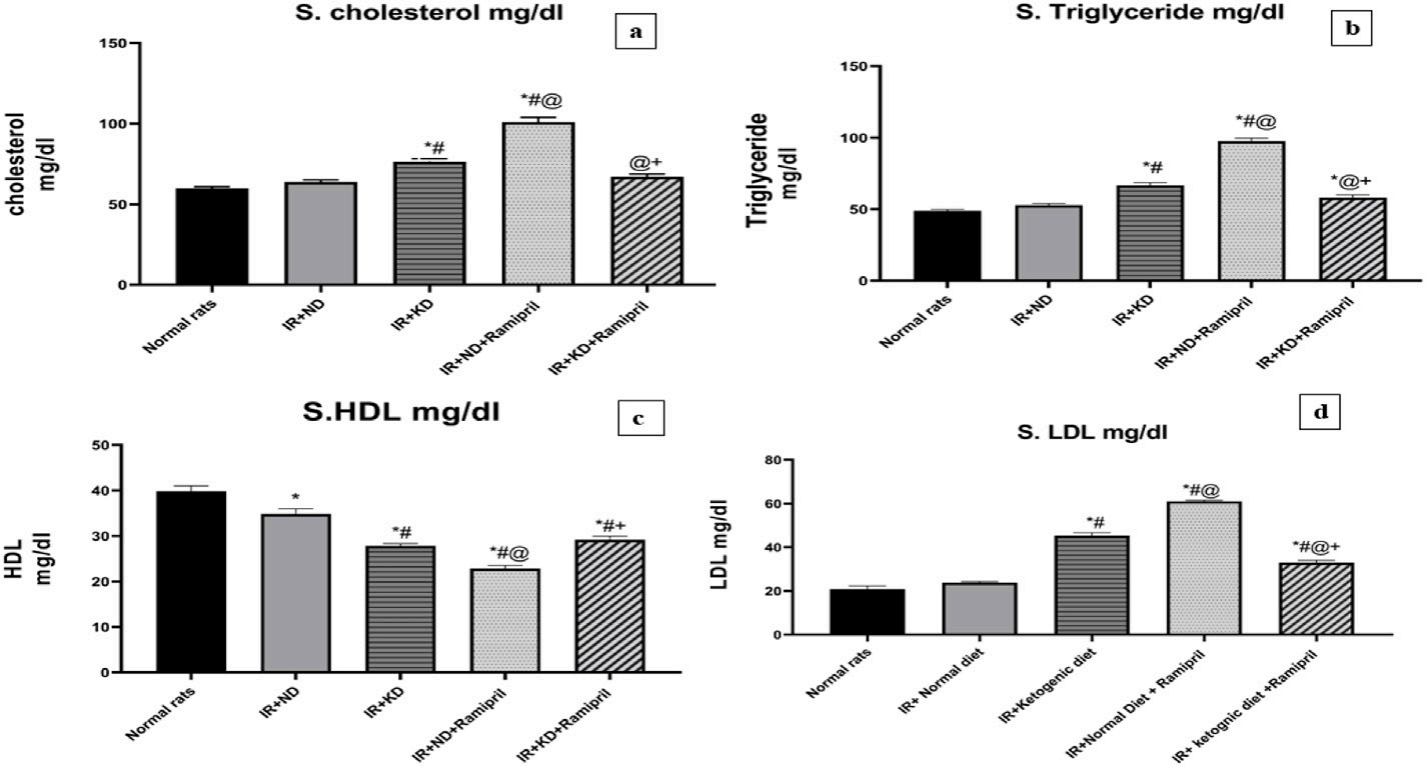
Effect of the ND and KD either with or without ramipril (2 mg/kg/day, orally) on the serum lipid profile: **(a)** serum cholesterol, **(b)** triglycerides, **(c)** high-density lipoprotein (HDL), and **(d)** low-density lipoprotein (LDL) in IR rats. The IR + KD + ramipril group showed significant decreases in serum cholesterol, triglycerides, and LDL levels but an increase in the HDL level compared to the IR + ND + ramipril group. Records are analyzed using one-way ANOVA, followed by the Tukey–Kramer test, and represented as mean ± S.E (n = 6), at *p < 0.05 vs. normal rats, #p < 0.05 vs. IR + ND, ^@^p < 0.05 vs. IR + KD, and ^+^p < 0.05 vs. IR + ND + ramipril group.

Although the TG levels were significantly increased in the IR + ND group compared to those in the normal group, these were noticeably reduced compared to those in the IR + KD and ramipril groups. In contrast, HDL levels were markedly decreased in the IR + KD group compared to those in normal rats and IR + ND groups; however, HDL levels were significantly increased compared to those in the IR + ND + ramipril group. Finally, LDL levels were noticeably higher in the IR + ND + ramipril group than in the IR + ND, IR + KD, and IR + KD + ramipril groups (Figure 4).

### 3.6 Effect of ramipril and KD on the brain-derived neurotrophic factor

**FIGURE 5:**
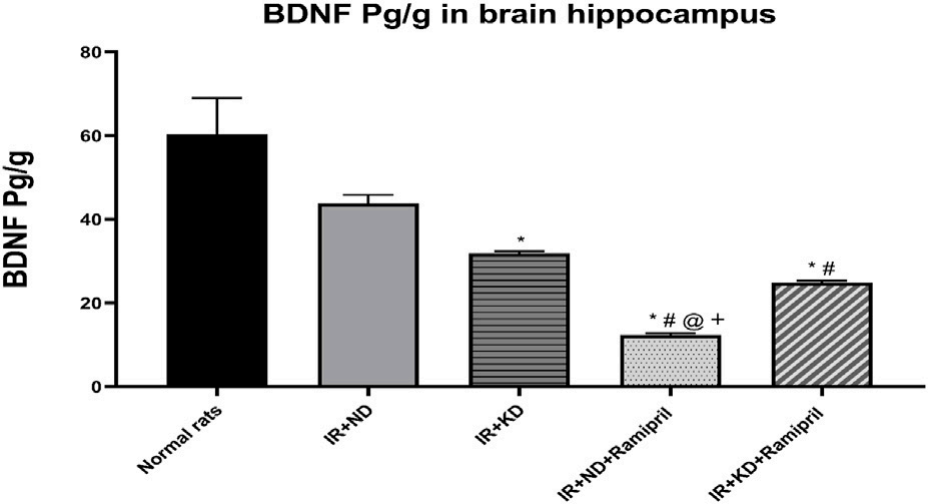
Effect of the ND and KD either with or without ramipril (2 mg/kg/day, orally) on the hippocampal brain-derived neurotrophic factor (BDNF) in IR rats. The IR + KD + ramipril group showed an increased level of hippocampal BDNF compared to the IR + ND + ramipril group, indicating that a KD enhanced the effect of ramipril. Records are analyzed using one-way ANOVA, followed by the Tukey–Kramer test, and represented as mean ± S.E (n = 6), at *p < 0.05 vs. normal rats, ^#^p < 0.05 vs. IR + ND, ^@^p < 0.05 vs. IR + KD, and +p < 0.05 vs. IR + ND + ramipril group.

The levels of BDNF were measured to study the effects of ramipril on IR-induced cognitive dysfunction in ND and KD mice. BDNF levels were reduced in the IR + KD, IR + ND + ramipril, and IR + KD + ramipril groups compared to those in normal rats. The IR + ND + ramipril group presented a noteworthy reduction in BDNF levels compared to all experimental groups. In the IR + KD + ramipril group, the BDNF levels were markedly reduced compared to those in the IR + ND group. BDNF levels were significantly higher in the IR + KD + ramipril group than in the IR + ND + ramipril group (Figure 5).

### 3.7 KD deteriorates the ramipril effect on glycogen synthase kinase-3 and insulin-degrading enzyme

**FIGURE 6:**
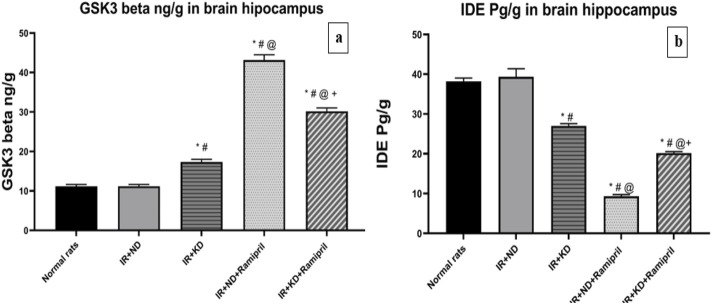
**(a,b)**. Effect of the ND and KD either with or without ramipril (2 mg/kg/day, orally) on **(a)** glycogen synthase kinase-3 beta (GSK3-β) and **(b)** insulin-degrading enzyme (IDE) in the brain hippocampus of IR rats. The IR + KD + ramipril group showed an increased level of hippocampal IDE and a decreased level of GSK3-β compared to the IR + ND + ramipril group, indicating that a KD enhanced the effect of ramipril. Records are analyzed using one-way ANOVA, followed by the Tukey–Kramer test, and represented as mean ± S.E (n = 6), at *p < 0.05 vs. normal rats, #p < 0.05 vs. IR + ND, ^@^p < 0.05 vs. IR + KD, and ^+^p < 0.05 vs. IR + ND + ramipril group.

GSK3β is the primary regulator of glucose metabolism in skeletal muscles and insulin regulation (Wang et al., 2022), whereas IDE is the major enzyme responsible for insulin and Aβ degradation (Tian et al., 2023). It has been implicated in IR development. GSK3β activity was significantly increased, whereas that of IDE was markedly decreased in the IR + KD, IR + ND + ramipril, and IR + KD + ramipril groups compared to the normal rats and IR + ND groups due to uncontrolled IR. The GSK3β activity was significantly higher, whereas that of IDE was markedly lower in both ramipril-treated groups than in IR + KD groups. The GSK3β activity was significantly lower, whereas that of IDE was considerably higher in the IR + KD + ramipril group than in the IR + ND + ramipril group (Figures 6a,b).

### 3.8 Ramipril and KD modulate hippocampus and cerebral cortex histopathological scores

**FIGURE 7:**
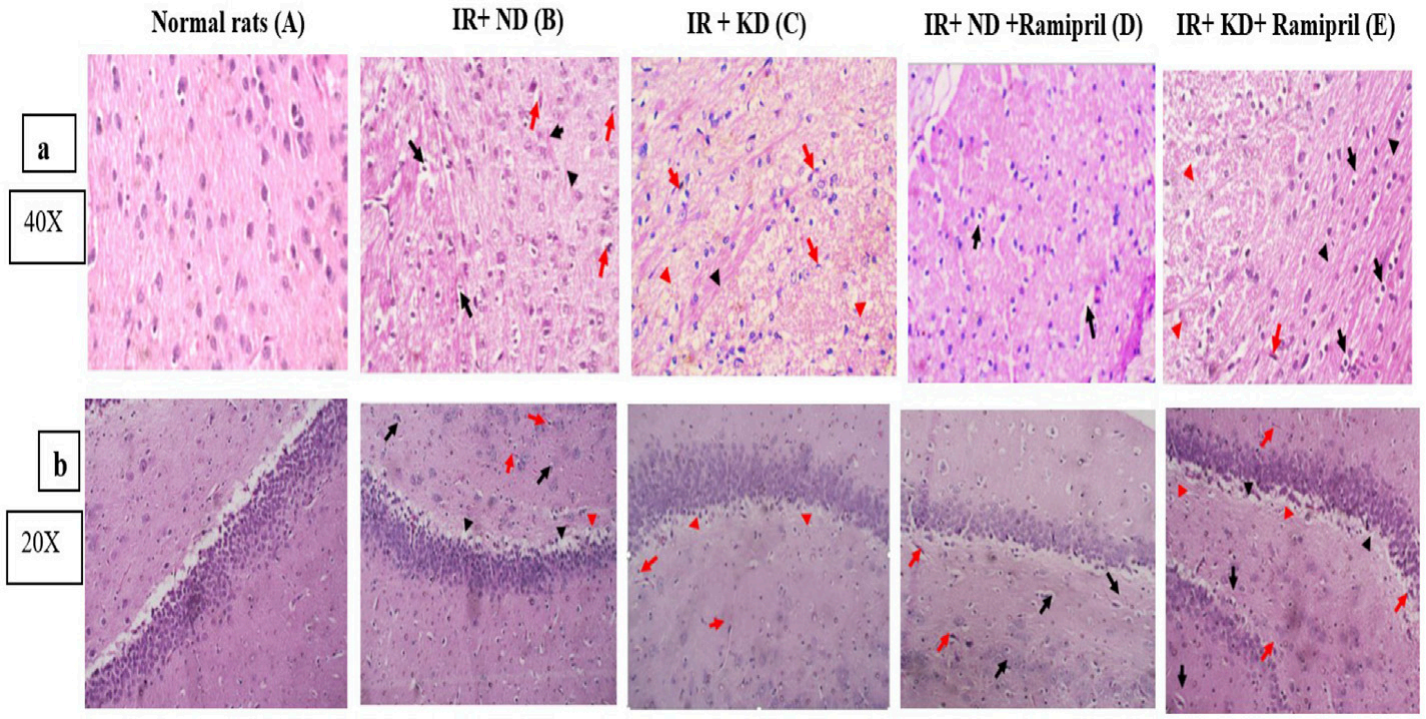
**(a,b)**. Effect of the ND and KD either with or without ramipril (2 mg/kg/day, orally) on histopathological examinations of insulin-resistant rats. **(a)** Cerebral cortex and **(b)** hippocampus. Photomicrographs of cerebral cortex slices stained with hematoxylin and eosin from various research groups. For normal rats in both brain tissues, **(A)** brain tissues were uniform and showed no evidence of neuronal damage. 0 grade for both tissues. **(B)** Neurons with pyknotic nuclei and peri-neuronal edema were present (black arrows). There were red neurons (shown by red arrows). There were gliosis regions in both tissues but with Rosenthal fibers in the cerebral cortex (black arrowheads). 75% of brain tissue… Grade 3 for both tissues. **(C)** There were a few red neurons dispersed throughout (red arrows), and tissue edema (red arrowheads) for both tissues and gliosis (black arrowheads) were observed for the cerebral cortex. 25% of brain tissue… Grade 2 for both tissues. **(D)** Unlike the cerebral cortex, the hippocampus showed few red neurons (red arrows), neurons with pyknotic nuclei, and perineuronal edema (black arrows). Cerebral cortex tissue showed mild perineuronal edema (black arrows). Both of them have no evidence of gliosis. 10% of brain tissues. **(E)** Both tissues shared similar features in the presence of very few scattered red neurons (red arrow), evidence of perineuronal edema (black arrows), tissue edema (red arrowheads), and areas of gliosis (black arrowheads). 20% of brain tissue… Grade 2. Images were taken at magnification power ×40 for the cerebral cortex, and ×20 for the hippocampus.

A 4-point scale was used to rate the severity of hippocampus and cerebral cortex microscopic lesions depending on the quantity and amount of tissue injury as follows: no lesions were found (grade 0). In minimal lesions (grade 1), the severity affected less than 15% of the field. In cases of mild (grade 2) injuries, 15%–45% of the tissue slices were affected. Approximately 45%–75% of the tissue segments were affected by moderate (grade 3) lesions. More than 75% of the tissue segments had noticeable (grade 4) lesions. Pathogenic alterations in the neural tissues were examined for red neurons, tissue edema, perineuronal edema, neuronal pyknosis, neuronophagia of degenerated neurons, necrosis, and reactive gliosis. Photomicrographs of the hippocampus and cerebral cortex from several groups are shown in Figures 7a,b, respectively. The hippocampal and cerebral cortex tissues of normal rats were homogenous and showed no signs of neuronal degeneration. Insulin-resistant rats in the ND group had neuronal damage, including red neurons, perineuronal edema, neurons with pyknotic nuclei, and gliotic regions within the Rosenthal fibers. Seventy-five percent of brain cells in the cerebral cortex scored grade 4, whereas the hippocampus scored grade 3. However, tissue edema, red neurons, and gliosis were visible in insulin-resistant rats with the KD—approximately at 25% of the investigated brain tissues. Grade 2 was assigned to both areas in our study. Ramipril-treated, insulin-resistant rats on the ND had the modest perineuronal edema in the cerebral cortex; however, the hippocampus displayed pyknotic nuclei, peri-neural edema, and red neurons with no gliosis. Ten percent of the tissue (grade 1) was located in the hippocampus and cerebral cortex. Furthermore, insulin-resistant rats treated with ramipril and nourished with the KD showed neuronal damage. Neuronal damage included a few scattered red neurons, peri-neuronal edema, and tissue edema. Both tissues shared the same score for 20% of the brain specimens (grade 2) (Figures 7a,b).

### 3.9 Ramipril and the ketogenic diet mitigated the Aβ and tau protein levels

**FIGURE 8:**
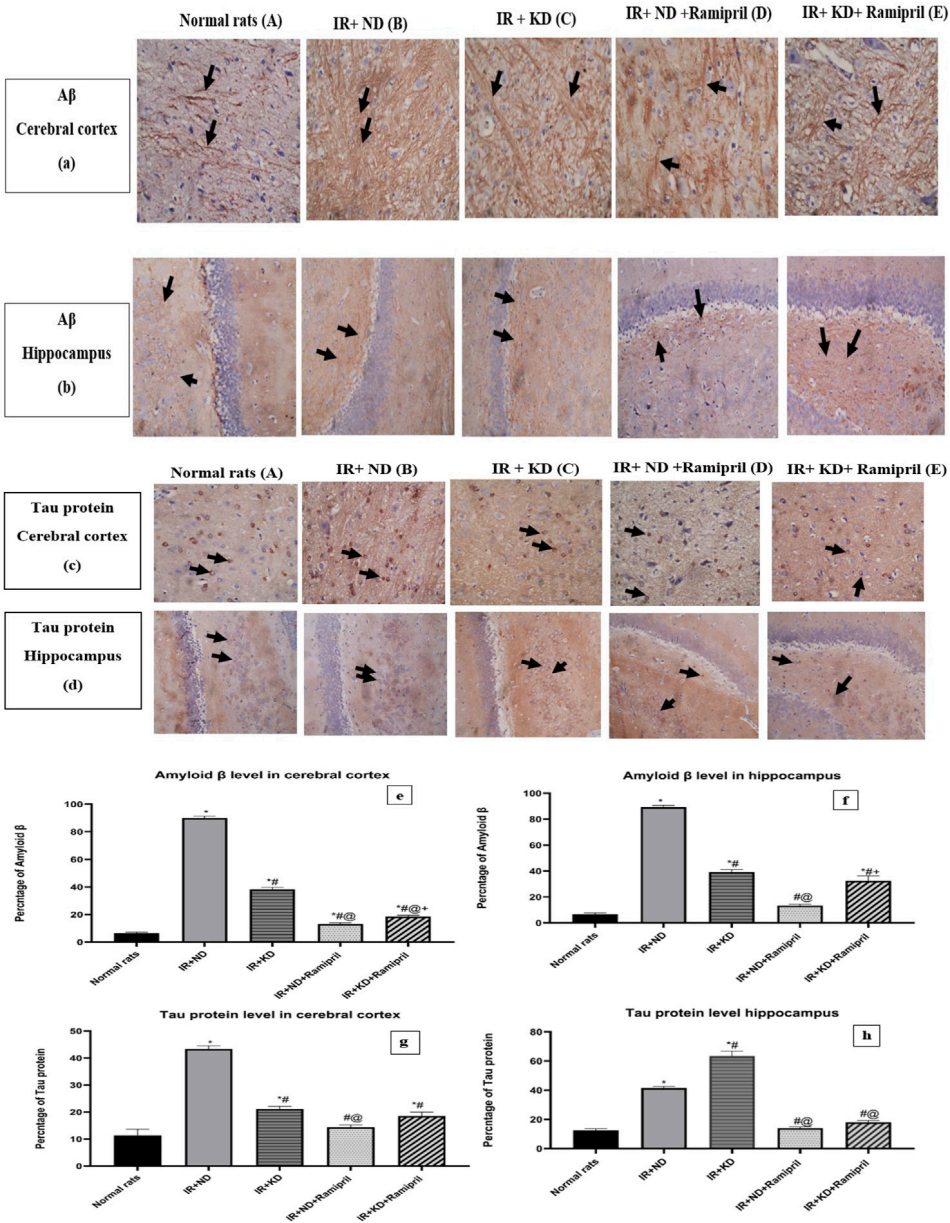
**(a)** Weak focal expression of Aβ (showed in the black arrows) in both brain tissues. **(b)** Marked increase in Aβ expressions (black arrows) in the cerebral cortex and hippocampus. **(c)** Marked reduction in Aβ expressions (black arrows) in both of them. **(d)** Weak focal expression of Aβ (black arrows) in the cerebral cortex and hippocampus. **(e)** Weak focal expression of Aβ (black arrows) in the cerebral cortex, whereas the hippocampus showed a marked increase in Aβ expression (black arrows). **(c,d)**. **(a)** Weak tau expression in the cytoplasm of a few neurons (black arrows) for both tissues. **(b)** Both the cerebral cortex and hippocampus showed an increase in the tau expression in many neurons (black arrows). **(c)** There is a reduction in the tau expression in many neurons (black arrows) in the cerebral cortex; conversely, the hippocampus exhibited an elevation in the expression of tau in many neurons (black arrows). **(d)** The cerebral cortex showed weak tau expression in the cytoplasm of a few neurons (black arrows), and hippocampus showed a mild elevation in the level of tau proteins in many neurons (black arrows). **(e)** Weak tau protein levels in the cytoplasm of some neurons for both tissues (black arrows). Images were taken at magnification power ×40 for cerebral cortex and ×20 for hippocampus. Statistical analysis showed the effect of the ND and KD with or without ramipril (2 mg/kg/day, orally) on immunohistochemical staining of the cerebral cortex and hippocampal levels of Aβ **(e,f)** and tau protein **(g,h)** of insulin-resistant rats. Records are analyzed using one-way ANOVA, followed by the Tukey–Kramer test, and represented as mean ± S.E (n = 6), at *p < 0.05 vs. normal rats; #p < 0.05 vs. IR + ND, ^@^p < 0.05 vs. IR + KD, and ^+^p < 0.05 vs. IR + ND + ramipril group.

The Aβ levels in the cerebral cortex were markedly observed in the IR + ND + ramipril group. All experimental groups presented a significant increase in Aβ levels compared to the normal group. Inversely, we noticed a marked reduction in Aβ levels in the two groups treated with ramipril and the IR + KD compared to the IR + ND group. In the case of the cerebral cortex, the addition of ramipril to the ND and KD significantly decreased Aβ expression compared to that in the IR + KD group; however, Aβ levels in the hippocampus were considerably reduced in the IR + ND + ramipril group compared to the IR + KD group. The cerebral cortex and hippocampus shared similar Aβ levels concerning the IR + KD + ramipril group as it was still noticeably high compared to those in the IR + ND + ramipril group (Figures 8a,b).

For the cerebral cortex, similar results were found at the tau protein levels as these were significantly increased in the IR + ND, IR + KD, and IR + KD + ramipril groups compared to normal rats. In contrast, the hippocampus displayed a noticeable increase in tau protein levels in the IR + ND and IR + KD groups compared to normal rats. A noticeable decrease was noted in tau protein levels in the cerebral cortex in the IR + KD group and the two rat groups that received ramipril compared to the IR + ND group. The hippocampus shared similar results with the cerebral cortex in all the previously mentioned groups, except for the IR + KD group, as it displayed an elevation compared to the IR + ND group. In the cerebral cortex tissue, the IR + ND + ramipril group exhibited a significant reduction compared to the IR + KD group. In the hippocampus, both groups treated with ramipril presented a significant decrease in tau protein levels in contrast to the IR + KD group (Figures 8c,d).

## 4 Discussion

Patients fed the KD depend on fat oxidation as an energy source. When the body experiences extreme hunger or a limited amount of carbohydrates, the liver transforms fatty acids into ketone bodies, which differs from pathological ketosis (Yuan et al., 2020). Long-term fructose consumption develops a state of hyperinsulinemia and hyperglycemia (Abdel-Kareem et al., 2024; Liu et al., 2022). We focused on how diet may affect the drug response in insulin resistance-induced cognitive dysfunction. Although different studies in the past few years have recommended the KD, more than one study in 2023 reported its harmful metabolic and non-metabolic effects.

Only switching from the rich fructose diet to the ND with moderate carbohydrate intake, as demonstrated in the present study, could improve insulin sensitivity (Al-Reshed et al., 2023; Barrea et al., 2023) and lipid profile levels. In contrast, the KD deteriorates insulin sensitivity because it is a stressor diet that increases cortisol levels through the stimulated hypothalamic pituitary adrenal axis due to dietary carbohydrate depletion (Barrea et al., 2023). Several studies have documented that KD exerts favorable effects on lipid profiles, and each study had its explanation (Schmidt et al., 2023; Yilmaz et al., 2021). We may expect that high cortisol levels, with the resulting hyperglycemia and hyperinsulinemia, play a crucial role in increased cholesterol, TG, and LDL levels, with reduced HDL levels. The failure of the KD group to achieve a decrease in body weight may be due to either overconsumption of fats or an imbalanced diet. However, ramipril treatment succeeded in achieving a noticeable decrease in body weight due to the inhibition of the renin–angiotensin–aldosterone system (RAAS) (Mitchell et al., 2021). This system inhibitor (ACEIs) enhances peroxisome proliferator-activated receptor gamma gene expression, a lipolytic gene in adipose tissues responsible not only for decreasing glucose, insulin, and TG levels, and body weight but also for increasing caloric expenditure (Santos et al., 2009).

Ramipril had no noteworthy effect on glucose metabolism in the nondiabetic population (Janka et al., 1990). Moreover, it inhibited ACE-1, which, in turn, inhibited the production of angiotensin II and decreased the production of angiotensin-(1–7) by ACE2. Recent studies have reported that angiotensin-(1–7) and bradykinin are inversely proportional to insulin resistance, as measured through HOMA-IR, and their low levels dysregulate the lipid profile (Cassis et al., 2019; Nozato and Yamamoto, 2021). Angiotensin-(1–7) enhances insulin sensitivity, glucose tolerance, and skeletal muscle glucose uptake and improves glucose metabolism in the adipose tissue (Zhao et al., 2021). Thus, ramipril administration worsened insulin resistance and the lipid profile in the IR + ND group treated with ramipril.

In contrast to the IR + ND + ramipril group, the IR + KD + ramipril group showed decreased insulin resistance, as measured through HOMA-IR. This may be explained as follows: the hypothalamic–pituitary–adrenal system was not activated dramatically due to the inhibition of the RAAS pathway by ramipril (Barrea et al., 2023). Although ramipril increases K^+^ levels (Chauhan et al., 2024), most studies have reported that the KD can reduce K^+^ levels as it restricts high-carbohydrate foods containing high K^+^ levels (Davis et al., 2008). This difference in the mechanism can create a balanced state at the K^+^ level. The relationship between potassium levels and insulin action is unclear; however, studies have demonstrated that the depletion of potassium impairs insulin action (Kim et al., 2015). Improved inulin sensitivity concurrently ameliorated the lipid profile. The correlation between ACE and glucose and fat metabolism is not fully understood, and further research is required to elucidate the exact mechanisms involved.

Although the ND mitigated both IR and the lipid profile peripherally, in addition to the central insulin sensitivity enhancement, proved by normalizing IDE and GSK-3β levels, the ND could not resolve either Aβ accumulation or the hyperphosphorylated tau protein. This implies that it did not improve IR-induced cognitive function, as was behaviorally proven using the MWM test.

Although the KD mitigated the Aβ and tau protein expressions, attenuating the IR-induced cognitive dysfunction, a negligible difference was noted in the MWM behavioral test in both phases between the groups. KD provides the brain with ketone bodies as an alternative fuel for glucose, enhances the genesis of new mitochondria, increases adenosine triphosphate production, produces fewer reactive oxygen species than glucose, decreases mitochondrial interaction of amyloid precursor protein, modifies gene expression associated with neurodegenerative diseases, and improves gene expression associated with metabolism in the hippocampus (Taylor et al., 2022). However, KD was unable to improve IDE or GSK-3β activity due to the untreated IR. In contrast, the IR + KD + ramipril group demonstrated false-negative results in the acquisition phase of the MWM short-term memory test as the rats spent a prolonged time to find the escape platform. This could be explained by the anxiety caused by KD-induced high cortisol levels, which seriously affected the concentration of animals, rather than their memory. Anxiety was observed during the MWM test, which was associated with elevated cortisol levels. These behaviors were detected in KD-fed rats. In brief, rats showed abnormal behaviors, such as swimming rapidly and jumping back into the water just after reaching the platform; these behaviors are believed to be due to stress.

Although the IR + ND + ramipril rats did not show improvement in the central IR, as indicated by decreased IDE activity and increased GSK-3β levels due to severe IR (Wei et al., 2021), they showed improvement in Aβ and tau accumulations as ACE enzyme inhibition prevented this accumulation. The contrasting results found in other studies could be attributed to genetic variability between the strains (Le et al., 2021).

The KD modulates the effect of ramipril on GSK3β, IDE, BDNF, and Aβ, and tau proteins compared to that of ND and ramipril together through unclear mechanisms. The KD depends on high-fat and low-carbohydrate intake. Low carbohydrate intake may affect ramipril action, making the KD more effective than the ND in supporting its action; further investigation is required to determine whether low carbohydrate levels play a role in RAAS suppression. Different studies have linked following a KD to decreased brain BDNF levels (Kackley et al., 2022; Vizuete et al., 2013; Gyorkos et al., 2019); further investigations are required to confirm these findings.

The ameliorative effects of ramipril and KD on hippocampus histopathological scores were parallel with the immunohistochemical outcomes of Aβ and tau accumulation. The relationship among Aβ, tau protein, and other metabolic markers remains elusive; further investigations are needed as existing studies have focused on the results of individual pathways. The link between these metabolic markers requires numerous efforts to elucidate how this combination may affect the disease status.

We suggest further investigations using this approach to shed light on the importance of diet in amplifying the therapeutic effects of drugs.

Similar to other drugs, the KD exhibited adverse effects. As both the KD and drugs share the ability to induce adverse effects, individuals continue to use drugs because of their therapeutic effects. Hence, similar to drugs, the KD should be prescribed based on a risk–benefit evaluation. KD should not be classified as a purely “good” or “bad” diet. Instead of this classification, we should understand patients’ disease status and diet outcomes by evaluating the potential benefits and risks.

We suggest applying the current proposal to a clinical trial to assess the outcomes of short- and long-term treatments.

One of the limitations of this study was the use of a minimum number of animals in each group (*n* = 6 per group) and reliance on a single animal model of IR in rats. Hence, future studies discussing similar topics should utilize a greater number of rats to increase the validity of the findings and use other models of IR to verify the findings. Another limitation was the absence of cortisol level measurements, which is recommended for future investigations involving the KD.

## Data Availability

The original contributions presented in the study are included in the article/supplementary material; further inquiries can be directed to the corresponding author.
